# Smoking and adipose tissue inflammation suppress leptin expression in Japanese obese males: potential mechanism of resistance to weight loss among Japanese obese smokers

**DOI:** 10.1186/1617-9625-10-3

**Published:** 2012-02-28

**Authors:** Shintaro Nagayasu, Shigeki Suzuki, Akiko Yamashita, Ataru Taniguchi, Mitsuo Fukushima, Yoshikatsu Nakai, Kazuko Nin, Naoya Watanabe, Shoichiro Nagasaka, Daisuke Yabe, Fusanori Nishimura

**Affiliations:** 1 Department of Dental Science for Health Promotion, Hiroshima University Graduate School of Biomedical Sciences, 1-2-3 Kasumi, Minami-ku, 734-8553 Hiroshima, Japan; 2 Division of Diabetes and Endocrinology, Kyoto Preventive Medical Center, 28 Nishinokyo, Samaryocho, Nakagyo-ku, Kyoto 604-8091, Japan; 3 Division of Clinical Nutrition and Internal Medicine, Okayama Prefectural University, 111 Kuboki, Soja-city, Okayama 719-1197, Japan; 4 Kyoto Institute of Health Science, Karasumaoike-Higashi, Nakagyo-ku, Kyoto 604-0845, Japan; 5 Human health Sciences, Graduate School of medicine, Kyoto University, Yoshida-Konoe-cho, Sakyo-ku, Kyoto 606-8501, Japan; 6 Health Care and Promotion Center, Yodogawa Christian Hospital, 2-9-26 Awaji, Higashiyodogawa-ku, Osaka 533-0032, Japan; 7 Division of Endocrinology and Metabolism, Department of Medicine, Jichi Medical University, 3311-1 Yakushiji, Shimotsuke-city 329-0498, Japan; 8 Division of Diabetes, Clinical Nutrition and Endocrinology, Kansai Electric Power Hospital, 2-1-7 Fukushima, Fukushima-ku, Osaka 553-0003, Japan

**Keywords:** Leptin, Smoking, Low-grade inflammation, Nicotine, ICAM-1

## Abstract

**Background:**

The effect of smoking on leptin regulation is controversial. Smoking may induce low-grade inflammation. Recent series of studies indicated the critical role of macrophage migration in the establishment of adipose tissue inflammation. In this study, we aimed to see the effects of smoking and inflammation on leptin regulation both at cellular and epidemiological levels.

**Methods:**

We compared the concentration of inflammatory markers and serum leptin levels among Japanese male subjects. Additionally, leptin and intercellular adhesion molecule (ICAM) -1 gene expression was assessed in adipocytes co-cultured with or without macrophages in the presence or absence of nicotine and/or lipopolysaccharide (LPS).

**Results:**

In subjects with BMI below 25 kg/m^2^, both WBC counts and soluble-ICAM-1 levels are significantly higher in smokers than in non-smokers. However, leptin concentration did not differ according to smoking status. However, in subjects with BMI over 25 kg/m^2^, smokers exhibited significantly lower serum leptin level as well as higher WBC counts and s-ICAM-1 concentration as compared with non-smokers. Leptin gene expression was markedly suppressed in adipocytes co-cultured with macrophages than in adipocyte culture alone. Furthermore, nicotine further suppressed leptin gene expression. ICAM-1 gene expression was markedly up-regulated in adipocytes co-cultured with macrophages when stimulated with LPS.

**Conclusions:**

Adipose tissue inflammation appears to down-regulate leptin expression in adipose tissues. Nicotine further suppresses leptin expression. Thus, both smoking and inflammation may diminish leptin effect in obese subjects. Therefore, obese, but not normal weight, smokers might be more resistant to weight loss than non-smokers.

## Background

Although there is no doubt that overall serum leptin concentration increases with increased body mass index and body fat content, there also observed large inter-individual differences in circulating leptin concentration even among obese subjects [1]. This may indicate production of leptin protein is regulated by various factors both at genetic and environmental levels. Among environmental factors, smoking appears to be one of such environmental factors, as smoking and its cessation have often been reported to be associated with low body mass index and weight gain [2]. However, previous reports on the effects of smoking on leptin levels differ from study to study, the one reporting increased leptin levels among smokers than in non-smokers and another with opposite results [3,4]. One of the reasons in such discrepancy may be in the fact that smoking may interact with other environmental factors such as host inflammatory responses and these interactions may further impair leptin regulations.

Recently, macrophages have been reported to be integrated into adipose tissues, thereby interacting with adipocytes, major source of circulating leptin, to exhibit higher inflammatory responses which greatly influences insulin resistance as well as increased cardiovascular risks [5,6]. Therefore, it is essential to understand leptin regulation according to adipose tissue maturation, inflammatory status as well as smoking status. Among several inflammatory markers, serum ICAM-1 level has recently been reported to positively associate with circulating leptin level in hypercholesterolemic patients [7].

The purpose of the present study is, therefore, to 1) examine circulating leptin and s-ICAM-1 levels as a representative of inflammatory markers among Japanese male subjects with or without smoking habits, and, then, to 2) understand possible underlying mechanisms on differential leptin regulation under the differential environmental conditions to ultimately try to understand the pathophysiological nature of obesity among smokers.

## Methods

### Human study

Total 360 male subjects who visited Health Care and Promotion Center, Yodogawa Christian Hospital for the purpose of medical check-up between Apr 1, 2009 and Sep 30, 2010 were enrolled in the study. The study protocol was approved by the ethics committee of Yodogawa Christian Hospital and informed consents were obtained from every participant. All subjects received regular medical checkup such as measurement of body weight, height, blood pressure, serum HDL and LDL cholesterol, triglycerides, fasting blood glucose, glycosylated hemoglobin (HbA1c), insulin, white blood cell (WBC) counts, AST, ALT, γ-GTP levels, and high-sensitivity CRP (hsCRP) levels. In addition to these regular measurements, serum leptin and s-ICAM-1 concentration was measured by commercial immunoassay kit (Quantikine, human leptin and human sICAM-1/CD54, R&D, Minneapolis, MN, USA, respectively). The subjects were first divided into two groups based on the body mass index (BMI), the one with obese (OB: BMI > 25.0 kg/m^2^: n = 111) and non-obese (NOB: BMI < 25.0 kg/m^2^: n = 249). The subjects in each group were then stratified into two sub-groups based on the questionnaire for smoking: one with current smokers (OB-S: n = 23; NOB-S: n = 70) and the other with current non-smokers (OB-NS: n = 88; NOB-NS: n = 179). Thus, in each obese and non-obese group, each measurement was compared between current smokers and no-smokers.

### Cell culture

Mouse 3T3-L1 preadipocytes and the mouse macrophage cell line RAW264.7 were used as described [8]. Pre-adipocytes were maintained in a Dulbecco's modified Eagle's medium (DMEM) containing 10% fetal bovine serum, and were differentiated into mature adipocytes with 4.5 mM glucose, 1 mM insulin, 1 mM dexamethasone, and 0.5 mM 3-isobutyl-1-methylxanthine as described previously. Mature adipocytes and RAW264.7 were co-cultured in a transwell system (Corning Inc., Acton, MA) with a 0.4-μm porous membrane to separate the upper and lower chambers. Then, 1 × 10^5 ^differentiated 3T3-L1 cells were cultured in the lower chamber, while 5 × 10^4 ^RAW cells were cultured in the upper chamber.

### Gene expression analyses

The cells were stimulated with or without 1 ng/ml of *E. coli *LPS or with or without nicotine for indicated time period. ICAM-1 and leptin gene expression in adipocytes were evaluated by real-time PCR as described previously [9]. The primers used are as follows; ICAM-1 (forward: CGATCTTCCAGCTACCATCC, reverse: CTTCAGAGGCAGGAAACAGG), Leptin (forward: TCTCCGAGACCTCCTCCATCT, reverse: TTCCAGGACGCCATCCAG), GAPDH (forward: AATGTGTCCGTCGTGGATCTGA, reverse: GATGCCTGCTTCACCACCTTCT).

### Statistical analyses

The differences of the serum leptin, s-ICAM-1 concentration, WBC counts as well as other medical parameters between OB-S and OB-NS and between NOB-S and NOB-NS were determined by student *t*-test. Difference of ICAM-1 gene expression in adipocytes co-cultured with macrophages with or without LPS was determined by student *t*-test, while multiple comparison among different culture conditions in the presence or absence of nicotine were determined by ANOVA.

## Results

### Serum leptin concentration is lower, while s-ICAM-1 concentration and WBC counts are higher, among smokers than among non-smokers in obese (BMI > 25.0 kg/m^2^) Japanese males

We first compared the differences of the measurements between smokers and non-smokers in both obese and non-obese Japanese males. Obese smokers (OB-S) exhibited significantly higher triglyceride levels as compared with those in obese non-smokers (OB-NS) (134.5 ± 59.4 mg/dl for non-smoker vs. 170.5 ± 92.0 for smoker, *p * < 0.015). This difference was not evident in non-obese group, suggesting that, in obese smokers, lipolytic and/or lipogenetic conditions could be induced. Lipolysis is induced by inflammation. Therefore, we next compared inflammatory markers as WBC counts and serum s-ICAM-1 concentration between OB-S and OB-NS, and between NOB-S and NOB-NS. As in Table 1, both WBC counts and s-ICAM-1 concentration are higher in smokers than in non-smokers in both obese and non-obese group, suggesting that smoking may induce low-grade inflammation. We further compared serum leptin levels between smokers and non-smokers. Although there observed no significant difference in leptin concentration between smokers and non-smokers in non-obese subjects, obese smokers exhibited significantly lower serum leptin concentration as compared with obese non-smokers (*p *< 0.007).

**Table 1:** WBC counts, serum s-ICAM-1, and leptin concentrations in each group


**OB: BMI>25.0 kg/ml^2^**	**OB-NS: non-smoker (N = 88)**	**OB-S: smoker (N = 23)**	***p*-value**

age (years)	54.5 (8.8: 36-73)	51.9 (10.2: 38-81)	0.571
BMI (kg/m^2^)	27.3 (2.30)	26.6 (1.42)	0.964
WBC count (/μl)	5945 (1405.7)	7323 (1749.8)	0.001
s-ICAM-1 (ng/ml)	190.9 (62.8)	238.2 (60.5)	0.004
Leptin (ng/ml)	4.76 (3.47)	3.18 (1,79)	0.007
HDL cholesterol (mg/dl)	51.88 (10.37)	49 (15.72)	0.15
LDL-cholesterol (mg/dl)	126.33 (28.84)	117 (30.40)	0.09
triglycerides (mg/dl)	134.5 (59.39)	170.55 (59.39)	0.015
HbA1c (%)	5.33 (0.34)	5.39 (0.68)	0.27
insulin (μU/ml)	6.46 (3.99)	5.11 (2.32)	0.07
AST (mU/ml)	33.25 (43.45)	22.36 (7.88)	0.12
ALT (mU/ml)	37.78 (24.23)	27.5(10.68)	0.03
γ-GTP (mU/ml)	65.61 (45.65)	68.14 (76.51)	0.42
systolic blood pressure (mmHg)	128.63 (11.65)	121.18 (12.23)	0.005
diastolic blood pressure (mmHg)	80.43 (8.74)	76.36 (8.90)	0.03
hsCRP (mg/dl)	0.14 (0.22)	0.11 (0.08)	0.24

**NOB: BMI < 25.0 kg/m^2^**	**NOB-NS: non-smoker (N = 179)**	**NOB-S: smoker (N = 70)**	***p*-value**

age (years)	56.3 (10.2: 33-79)	55.6 (7.8: 36-75)	0.141
BMI (kg/m^2^)	22.2 (1.79)	22.1 (1.1)	0.123
WBC count (/μl)	5560 (1339.2)	6690 (1662.8)	< 0.001
s-ICAM-1 (ng/ml)	172.6 (58.6)	219.5 (92.1)	< 0.001
Leptin (ng/ml)	2.37 (1.35)	2.32 (1.01)	0.744
HDL cholesterol (mg/dl)	61.94 (13.39)	58.04 (16.56)	0.03
LDL-cholesterol (mg/dl)	113.84 (29.43)	113.97 (31.35)	0.49
triglycerides (mg/dl)	105.60 (116.82)	116.14 (69.46)	0.24
HbA1c (%)	5.23 (0.36)	5.22 (0.31)	0.45
insulin (μU/ml)	3.4 (1.76)	3.72 (2.84)	0.14
AST (mU/ml	22.92 (7.04)	20.75 (5.55)	0.01
ALT (mU/ml)	22.76 (10.93)	19.78 (7.69)	0.02
g-GTP (mU/ml)	43.78 (37.6)	47.73 (35.83)	0.23
systolic blood pressure (mmHg)	119.69 (12.46)	117.81 (13.36)	0.15
diastolic blood pressure (mmHg)	74.55 (8.32)	74.51 (8.54)	0.49
hsCRP (mg/dl)	0.09 (0.2)	0.11 (0.15)	0.26

Means and (SD) are shown. Range for age is also shown within parenthesis.

### ICAM-1 gene expression in adipocytes is markedly up-regulated in adipocytes co-cultured with macrophages stimulated with LPS

**Figure 1:**
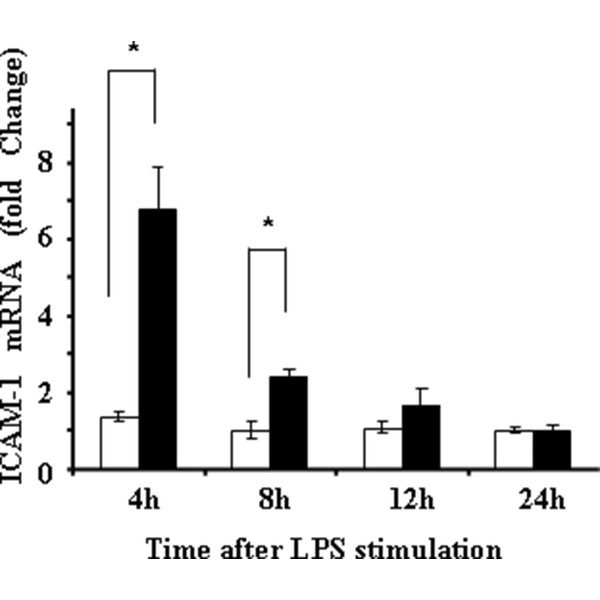
**ICAM-1 gene expression in adipocytes co-cultured with macrophages with (black bar) or without (white bar) LPS stimulation**. ICAM-1 gene expression was evaluated by real-time PCR, and the data are expressed as fold change of the levels at 0 h. *p < 0.05.

We previously reported that ICAM-1 gene expression was markedly up-regulated in adipocytes co-cultured with macrophages in the presence of LPS than in adipocytes co-cultured with macrophages without LPS stimulation by DNA microarray analyses [9]. We therefore confirmed these results by real-time PCR. As in Figure 1, at 4 and 8 h following 1 ng/ml of LPS stimulation, ICAM-1 mRNA levels were markedly up-regulated, suggesting that inflammatory conditions up-regulated ICAM-1 expression in adipocytes.

### Leptin gene expression is down-regulated in adipocytes co-cultured with macrophages, and further suppressed by LPS or nicotine

**Figure 2:**
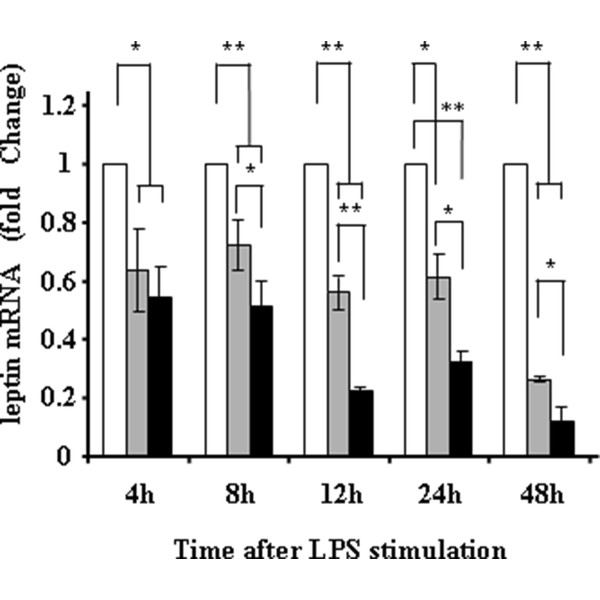
**Leptin gene expression in adipocytes without co-culture (white bar), co-cultured with macrophages (gray bar), and co-cultured with macrophages in the presence of 1 ng/ml of LPS**. Leptin gene expression was evaluated by real-time PCR. The data are expressed as fold changes of baseline data (expression at 0 h). *p < 0.05, **p < 0.001.

**Figure 3:**
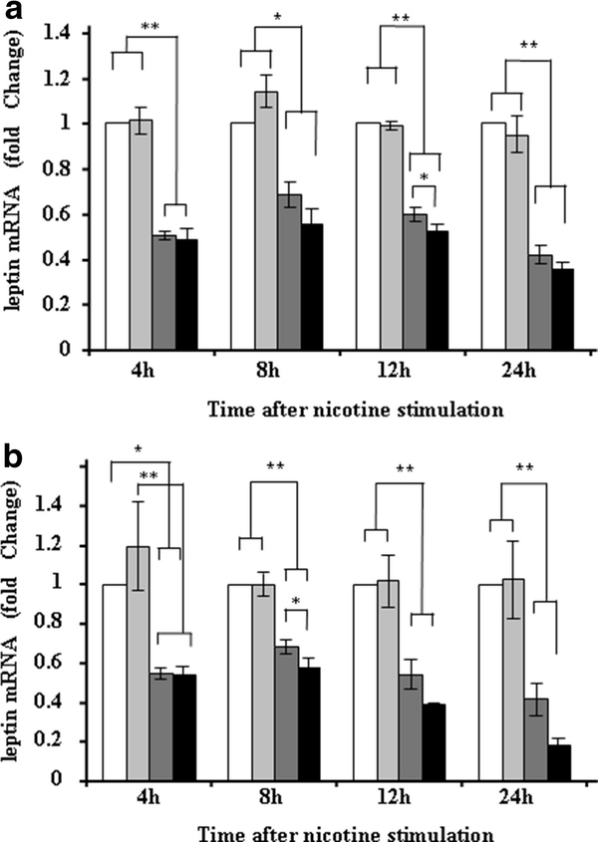
**Leptin gene expression in adipocytes single culture without nicotine (white bar) or with nicotine (pale gray bar), and in adipocytes co-cultured with macrophages in the absence of nicotine (dark gray bar) and in the presence of nicotine (black bar)**. (A) One μM of nicotine was used for experiments. Co-culture with macrophages greatly suppressed leptin gene expression in adipocytes. Addition of nicotine further suppressed leptin gene expression after 12 h of stimulation. The data are expressed as fold changes of baseline data (expression at 0 h). *p < 0.05, **p < 0.001. (B) Hundred μM of nicotine was used for the experiments. In this condition, nicotine further suppressed leptin gene expression at 8 h after nicotine stimulation which was markedly suppressed when the adipocytes were co-cultured with macrophages. However, nicotine did not directly suppress leptin gene expression in adipocytes. The data are expressed as fold changes of baseline data (expression at 0 h). *p < 0.05, **p < 0.001.

Since epidemiological observation suggested that smoking and inflammation appeared to suppress serum leptin levels in obese subjects, we are interested to see the involvement of macrophage on leptin gene expression and the effects of LPS or nicotine. As in Figures 2 and 3, leptin expression was significantly suppressed in adipocytes co-cultured with macrophages, and further suppressed in the presence of 1 ng/ml of LPS and 1 μM, 100 μM of nicotine. Although 10 nM of nicotine showed a trend suppressing leptin gene expression in adipocytes co-cultured with macrophages, the difference was not statistically significant (data not shown). Since there observed no direct suppressive effects of nicotine on adipocyte expression of leptin (Figure 3), the suppressive effects of leptin gene expression by nicotine appeared to be mediated *via *macrophages.

## Discussion

In this study, we first carefully checked the result of regular medical checkup, and found that obese male smokers exhibited higher WBC counts and s-ICAM-1 levels as well as higher triglyceride levels, suggesting that inflammation may up-regulates triglyceride levels. We selected only male subjects for the analysis, as there are very few female smokers in the middle aged population in Japan. Inflammation is closely linked with high triglyceride levels. One of the major adipokines, tumor necrosis factor (TNF)-α, is known to induce lipolysis [10]. Therefore, we hypothesized that adipose-tissue inflammation-derived TNF-α may be associated with elevated levels of triglycerides, as following lipolysis, released free fatty acids are integrated into the liver and triglycerides are re-synthesized afterwards [11]. In fact, we already confirmed that cultured 3T3-L1 adipocytes exhibited higher lipolytic activity when co-cultured with macrophages in the presence of TNF-α- inducing molecule as bacterial endotoxin [11]. We divided the subjects according to the subjects' BMI into two groups, obese and non-obese groups. In Japan, prevalence of the subjects with BMI over 30 kg/m^2 ^is estimated to be no more than 3% of all populations. However, it is also reported that in Japanese obese subjects with BMI over 25 kg/m^2^, although subcutaneous adipose tissue content is far less than that of Caucasians, visceral adipose tissue content does not remarkably differ from that of Western populations with BMI over 30 kg/m^2 ^[12]. This may suggest that visceral fat plays important role characterizing Japanese obesity. Additionally, interaction between infiltrated macrophages and adipocytes has been suggested to be associated with patho-physiology of many obesity-related dysfunctions such as enhanced insulin resistance, vicious cycle of adipose tissue inflammation, and subsequent augmentation of cardiovascular risks [13].

In this context, we previously performed gene expression analysis comparing gene expression profile in adipocytes co-cultured with macrophages with or without LPS stimulation by microarray technique, and found that LPS markedly up-regulated ICAM-1 gene expression in adipocytes [9]. In that study, ICAM-1 gene expression was 20.9 times higher at 4 h after LPS stimulation, 5.6 times higher at 8 h, 1.5 times higher at 12 h, respectively, and returned to basal level at 24 h (0.3 fold expression of that at 0 h) [9]. Our current real-time PCR analysis well confirmed the results of previously performed microarray analysis. Since s-ICAM-1 concentration was higher in smokers than in non-smokers in both obese and non-obese groups, one can claim that the major source of s-ICAM-1 could be non-adipose tissues such as endothelium. This speculation might be reasonable, as endothelium is directly exposed to circulating nicotine. Nevertheless, s-ICAM-1 concentration was higher in obese smokers than in non-obese smokers. Thus, we speculate that adipose tissue-derived s-ICAM-1 exhibit additive effects on circulating s-ICAM-1 originally released from non-adipose tissue, both of which further up-regulates cardiovascular risk in smokers. In obese smokers, WBC counts and s-ICAM-1 concentration were higher than non-smokers, while leptin concentration was significantly lower in obese smokers. These observations led us to investigate the effects of inflammation and nicotine on leptin gene expression in a similar way. The results indicated that adipocyte expression of leptin gene was markedly suppressed in the presence of macrophages than adipocyte single culture alone, and both LPS and nicotine further suppressed leptin gene expression. The suppressive effect of nicotine on leptin gene expression appeared to be mediated by macrophages, as there observed no direct suppressive effect of nicotine on adipocyte single culture. In fact, it has been documented that macrophages express nicotine receptor on the cell surface [14]. Based on these in vivo and in vitro observations, we speculate that adipocyte production of leptin is inhibited only in obese smokers but not in normal weight smokers and in non-smokers. Of course it is still possible that the suppressive effect of nicotine on leptin expression may not be fully due to direct effects of nicotine. We think 100 μM of nicotine is very high as compared with reported serum nicotine concentration in smokers, which is around at 10 μM [15]. However, although not statistically significant, we observed a trend that 1 μM of nicotine also suppressed leptin gene expression in co-culture model. Since co-culture itself without nicotine stimulation markedly suppressed leptin gene expression and WBC counts are higher in obese smokers than in non-smokers, we speculate some of the suppressive effects may be mediated by inflammation in obese smokers. In fact, it was previously shown that TNF-α inhibited leptin secretion from adipocytes [16]. Additionally, it is still possible that observed suppressive effects may be mediated by catecholamines as previously demonstrated [4]. Furthermore, the degree of smoking may also greatly influence the results. Further study is necessary to subdivide the subjects according to the degree of smoking as well as duration.

In this study, obese smokers exhibited lower leptin concentration as compared with non-smokers. Of course smokers may have different life style including intensity of exercise, food content, or sleep duration. Although we did not investigate sleep duration in our current study, the intensity of exercise, estimated by standard questionnaires, was not statistically significant between smokers and nonsmokers in obese or nonobese groups studied (data not shown). Higher leptin concentrations are related to the increased sympathetic nerve activity and hypertension in the obese subjects [17]. In fact, in this study, although we did not observe significant differences between smokers and non-smokers in non-obese group, both systolic and diastolic blood pressure were significantly lower in obese smokers with lower leptin levels as compared with obese non-smokers with higher leplin concentration.

It is generally believed that obese subjects are characterized by leptin resistance [18]. In fact, in this study, obese Japanese males exhibited higher serum leptin concentrations than non-obese males regardless the condition of smoking status. Yet obese smokers exhibited lower leptin levels than non-smokers. Recently, endoplasmic reticulum stress (ER stress) has been suggested to be linked with the molecular mechanisms of leptin resistance [19]. Interestingly, ER stress is also known to induce low-grade inflammation and, thus, is associated with insulin resistance [20]. More importantly, smoking may also induce ER stress in a variety of organs [21,22]. Moreover, if we assume obese smokers suffer from both leptin resistance, which was caused by the obesity, and insufficient leptin production, which was caused by low-grade inflammation and/or nicotine, these subjects might be more resistant to diet-therapy or to exercise-oriented weight loss. In fact, relationship between metabolic syndrome and smoking in Japanese population has been documented in a previous study [23].

## Conclusions

In summary, it can be concluded that smoking may suppress leptin expression in obese subjects via inflammation and nicotine-effect on adipose tissue. Therefore, obese, but not normal weight, smokers might be more resistant to weight loss than non-smokers.

## Competing interests

The authors declare that they have no competing interests.

## Authors' contributions

AT, MF, YN, KN, NW, SN, YD, SN, SS, contributed to data collection. SN, SS, and FN contributed to data analysis. SN, AT, and FN interpreted the data, and wrote the manuscript. All authors read and approved the final manuscript.
